# Novel image analysis approach for quantifying expression of nuclear proteins assessed by immunohistochemistry: application to measurement of oestrogen and progesterone receptor levels in breast cancer

**DOI:** 10.1186/bcr2187

**Published:** 2008-10-23

**Authors:** Elton Rexhepaj, Donal J Brennan, Peter Holloway, Elaine W Kay, Amanda H McCann, Goran Landberg, Michael J Duffy, Karin Jirstrom, William M Gallagher

**Affiliations:** 1 UCD School of Biomolecular and Biomedical Science, UCD Conway Institute, University College Dublin, Belfield, Dublin 4, Ireland; 2 UCD School of Medicine and Medical Science, UCD Conway Institute, University College Dublin, Dublin 4, Ireland; 3 Department of Histopathology, Education and Research Centre, The Royal College of Surgeons in Ireland, Beaumont Hospital, Dublin 9, Ireland; 4 Center for Molecular Pathology, Department of Laboratory Medicine, Malmö University Hospital, Lund University, Malmö, Sweden; 5 Department of Pathology and Laboratory Medicine, St Vincent's University Hospital, Dublin 4, Ireland

## Abstract

**Introduction:**

Manual interpretation of immunohistochemistry (IHC) is a subjective, time-consuming and variable process, with an inherent intra-observer and inter-observer variability. Automated image analysis approaches offer the possibility of developing rapid, uniform indicators of IHC staining. In the present article we describe the development of a novel approach for automatically quantifying oestrogen receptor (ER) and progesterone receptor (PR) protein expression assessed by IHC in primary breast cancer.

**Methods:**

Two cohorts of breast cancer patients (*n* = 743) were used in the study. Digital images of breast cancer tissue microarrays were captured using the Aperio ScanScope XT slide scanner (Aperio Technologies, Vista, CA, USA). Image analysis algorithms were developed using MatLab 7 (MathWorks, Apple Hill Drive, MA, USA). A fully automated nuclear algorithm was developed to discriminate tumour from normal tissue and to quantify ER and PR expression in both cohorts. Random forest clustering was employed to identify optimum thresholds for survival analysis.

**Results:**

The accuracy of the nuclear algorithm was initially confirmed by a histopathologist, who validated the output in 18 representative images. In these 18 samples, an excellent correlation was evident between the results obtained by manual and automated analysis (Spearman's ρ = 0.9, *P *< 0.001). Optimum thresholds for survival analysis were identified using random forest clustering. This revealed 7% positive tumour cells as the optimum threshold for the ER and 5% positive tumour cells for the PR. Moreover, a 7% cutoff level for the ER predicted a better response to tamoxifen than the currently used 10% threshold. Finally, linear regression was employed to demonstrate a more homogeneous pattern of expression for the ER (*R *= 0.860) than for the PR (*R *= 0.681).

**Conclusions:**

In summary, we present data on the automated quantification of the ER and the PR in 743 primary breast tumours using a novel unsupervised image analysis algorithm. This novel approach provides a useful tool for the quantification of biomarkers on tissue specimens, as well as for objective identification of appropriate cutoff thresholds for biomarker positivity. It also offers the potential to identify proteins with a homogeneous pattern of expression.

## Introduction

The oestrogen receptor (ER) remains the only reliable predictor of endocrine responsiveness in breast cancer, and is arguably the single most important predictive biomarker in clinical oncology today [1]. Moreover, one of the most studied ER-regulated genes is the progesterone receptor (PR). Approximately 70% to 80% of all invasive breast cancers are ER-positive and thus are considered likely to respond to endocrine therapy. The PR, which is positive in approximately 60% of cases, may be even more important in predicting response to anti-oestrogens [2].

Both premenopausal and postmenopausal women benefit from 5 years of treatment with the anti-oestrogen tamoxifen [3]. Current treatment guidelines for premenopausal women with hormone-responsive breast cancer advocate a combination of ovarian ablation/suppression or chemotherapy, followed by 5 years of tamoxifen treatment [4-6]. In hormone-responsive postmenopausal women, data from large prospective randomised controlled trials involving aromatase inhibitors are now emerging and herald new standards in adjuvant endocrine treatment [7,8]. The International Expert Consensus on the Primary Therapy of Early Breast Cancer states that tamoxifen may be an acceptable option, but that aromatase inhibitors have shown superiority over tamoxifen in postmenopausal breast cancer [5].

Irrespective of their menopausal status, arguably the single most important issue for any breast cancer patient is the assessment of her tumour hormone receptor status. The hormone receptor status is routinely evaluated in all resected primary tumours to assess the levels of ER and PR. Inmmunohistochemistry (IHC) performed on formalin-fixed tissue sections is now the most commonly used assay, having replaced biochemical-based methods. The hormone receptor status is currently assessed by a pathologist; with a cutoff threshold of 10% positive tumour cells being commonly employed to predict responsiveness to adjuvant hormonal therapy. Such a threshold can lead to significant intra-observer variability. For example, one study of 172 German pathologists highlighted the difficulties that can arise from manual assessment, with 24% of ER staining interpreted as being falsely negative [9]. Improved image analysis technologies have the potential to circumvent the burden of interpretation and intra-observer variability, offering the potential to develop objective automated quantitative scoring models for IHC. A move away from the semiquantitative manual scoring models currently employed should lead to less variability in results, to increased throughput and to the identification of new prognostic subgroups, which may not have been evident following initial manual analysis alone [10].

In the present article we propose an automated approach, based on unsupervised learning, to accurately assess the ER and PR expression levels in an extensive cohort of breast cancer specimens. In particular, our approach employs a novel approach to the identification of tumour nuclei, whereby nontumour structures, including stromal components and lymphocytic infiltrate, are automatically excluded from any analysis. Such an approach should allow for more accurate assessment of the IHC signal.

## Materials and methods

### Patients and tumour samples

Two patient cohorts were used in the present study (Table 1). The studies were approved by the ethical committees at Lund University and Linköping University.

**Table 1:** Clinicopathological characteristics of Cohorts I and II

	Cohort I (*n* = 179)	Cohort II (*n* = 564)
Age (years)		
Median (range)	65 (35 to 97)	45 (25 to 57)
Tumour size		
≤ 20 mm	105 (59)	208 (37)
>20 mm	74 (41)	356 (63)
Grade		
I	38 (21)	58 (10)
II	79 (44)	222 (40)
III	62 (35)	234 (42)
Missing		50 (8.9)
Histological type		
Indeterminate	3 (2)	7 (2)
Invasive ductal carcinoma	125 (70)	411 (73)
Invasive lobular carcinoma	31 (17.3)	43 (7.6)
Tubular	16 (8.9)	5 (1)
Medullary	4 (2.2)	25 (4)
Mucinous		3 (1)
Missing		70 (12)
Nodal status		
Negative	87 (49)	160 (28)
Positive	65 (36)	402 (71)
Missing	27 (15)	2 (0.3)
Oestrogen receptor status		
Positive	157 (88)	324 (57)
Negative	22 (12)	151 (27)
Missing		89 (16)
Progesterone receptor status		
Positive	52(29)	147 (26)
Negative	127 (71)	312 (55)
Missing		105 (19)

Data in parentheses represent percentages unless otherwise stated.

Cohort I (test cohort) consisted of 179 consecutive cases of invasive breast cancer diagnosed at the Department of Pathology, Malmö University Hospital, Malmö, Sweden, between 2001 and 2002. The median age at diagnosis was 65 years (range 35–97 years) and the median follow-up period for overall survival (OS) was 52 months (range 4–63 months).

Cohort II (validation cohort) consisted of 564 premenopausal women with primary breast cancer from the south and southeast regions of Sweden who enrolled in a multicentre clinical trial between 1984 and 1991. Patients were randomly assigned to either 2 years of adjuvant tamoxifen treatment (*n* = 276) or to a control group (*n* = 288); the aim of this study was to examine the effect of tamoxifen on recurrence-free survival (RFS) [2].

### Tissue microarrays and immunohistochemistry

Tissue microarrays (TMAs) were constructed using either a manual tissue arrayer (MTA-1; Beecher Inc., Sun Prairie, WI, USA) (Cohort I) or an automated tissue arrayer (ATA-27; Beecher Inc.) (Cohort II). Two 1.0 mm cores (Cohort I) or two 0.6 mm cores (Cohort II) were extracted from each donor block and were assembled in a recipient block as previously described [11]. IHC analysis was performed on 4 μm sections in the Ventana Benchmark system (Ventana Medical Systems Inc., Tucson, AZ, USA) using prediluted antibodies to the ER (anti-ER, clone 6F11; Ventana) or to the PR (anti-PR, clone 16; Ventana) as previously described [11]. 3,3'-Diaminobenzidine (DAB) was used as a chromogenic substrate, and the slides were counterstained using haematoxylin.

### Image acquisition, management and analysis

**Figure 1:**
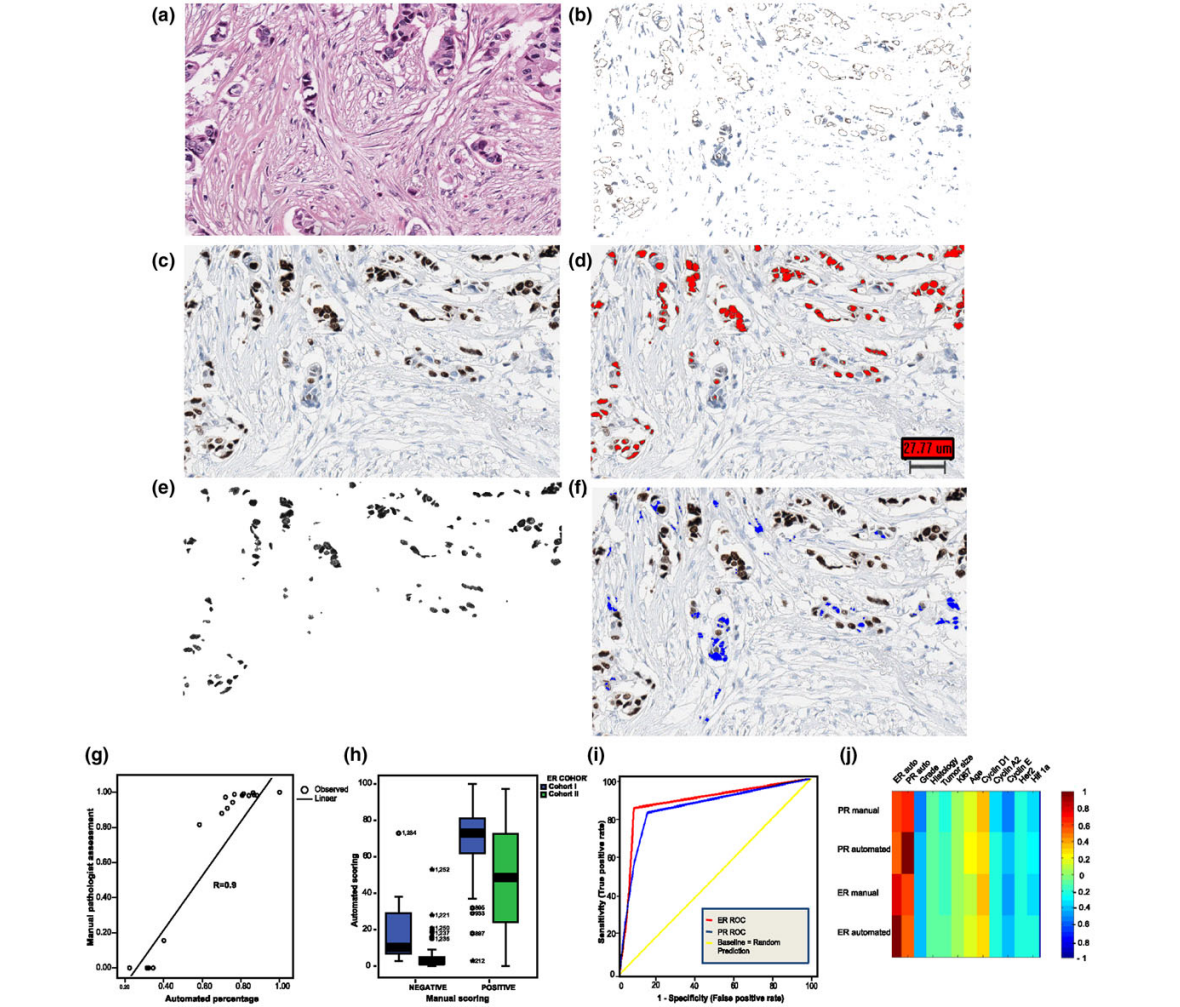
**Overview of the automated image analysis process and correlation between automated and manual analysis**. An example 500 × 500 pixel image taken to demonstrate the stepwise image process underlying the nuclear algorithm. **(a) **Original immunohistochemistry (IHC) section and **(b) **the equivalent H&E section. **(c) **Original IHC section after the extraction of 3,3'-diaminobenzidine (DAB)-positive tumour nuclei and **(d) **after the removal of DAB-negative tumour nuclei. **(e) **Identification (red) of DAB-positive tumour nuclei. **(f) **Identification (blue) of DAB-negative tumour nuclei. A more detailed description of the algorithm is available in Additional files 1 and 2. **(g) **Scatter plot demonstrating strong correlation between automated scores and manual annotation of the same cores by a pathologist. **(h) **Box plot (median, 25th and 75th quartiles) demonstrating the distribution of the oestrogen receptor (ER) quantitative automated data in relation to manual analysis in both cohorts. **(i) **Receiver-operator curves (ROCs) for the ER and the progesterone receptor (PR), with the number of false positives plotted along the abscissa and the number of true positives plotted along the ordinate (a curve more to the upper-left corner implies better performance). **(j) **Heat map showing the correlation between ER and PR expression determined by both automated and manual analysis and a number of clinicopathological parameters.

Digital images were captured using the Aperio ScanScope XT Slide Scanner (Aperio Technologies, Vista, CA, USA) as previously described [10]. Figure 1a to 1f outline a schematic representation of the algorithm output, and a full description is available in Additional files 1 and 2.

### Algorithm development and statistical analysis

Algorithms were developed using MatLab 7 (MathWorks, Apple Hill Drive, MA, USA). Statistical analysis was carried out using MatLab 7 (MathWorks) and SPSS version 11.0 (SPSS Inc., Chicago, IL, USA). Spearman's ρ correlation was used to estimate the relationship between automated and manual analysis. Univariate Cox regression analysis, Kaplan–Meier analysis and the log-rank test were used to illustrate differences between the RFS, OS and breast cancer-specific survival (BCSS) according to the expression of ER and PR. All *P *values are two-sided, and *P *< 0.05 was considered statistically significant.

## Results

The present study involved the development of an automated unsupervised nuclear algorithm to objectively assess the ER and PR expression levels in 743 breast tumours. Two breast cancer cohorts (*n* = 743) as described above were used (Table 1). Manual ER and PR expression data were available for 654 (88%) patients and 640 (86%) patients, respectively. As a number of cores were not suitable for image analysis due to large image artefacts, automated analysis was restricted to 639 patients (86%) for whom ER data were available and to 622 (84%) patients for whom PR data were available.

### Quantitative assessment of ER and PR status: correlation between manual and automated approaches

Nuclear ER and PR expression levels were quantitatively determined using the aforementioned algorithm (Figure 1a to 1f). The accuracy of the algorithm was confirmed by a histopathologist (PH), who validated the output in 18 representative ER images. An excellent correlation was evident between the percentage positive tumour nuclei as determined by image analysis compared with determined by manual analysis (Spearman's ρ = 0.9, *P *< 0.001) (Figure 1g). The estimated misclassification rate of nuclei (tumour versus nontumour) was < 10%, thus confirming the ability of the algorithm to identify tumour from stromal tissue and also confirming the accuracy of the algorithm in quantifying ER expression.

Following this initial validation stage, the ER and PR expression levels as determined by image analysis were compared with the manual assessment of the two cohorts outlined above. Figure 1h illustrates the distribution of ER expression levels as determined by image analysis, and demonstrates a distinct separation between the ER-negative and the ER-positive groups in both cohorts. Similar results were evident for the PR (data not shown). Once again, a strong correlation between image analysis and manual assessment was evident for both the ER (Spearman's ρ = 0.74, *P *< 0.001) and the PR (Spearman's ρ = 0.62, *P *< 0.001). The algorithm was particularly impressive in borderline cases (that is, cases manually annotated as 1% to 10% positive), where an accuracy level of 90% was evident. Receiver-operator curve analysis was also performed comparing manually and automatically assessed ER and PR expression levels (Figure 1i). The area under the curve was excellent for both the ER (area under the curve = 0.85) and the PR (area under the curve = 0.74), further confirming the accuracy of the automated algorithm.

As breast cancer is a heterogeneous disease with different histological subtypes and grades, the correlation between a number of clinicopathological parameters and both manual and automated assessment was examined. Figure 1j illustrates that similar correlations were seen between the ER and PR expression and histological subtypes, grade, tumour size and a number of other IHC markers irrespective of the assessment method employed (manual versus automated). Figure 1j demonstrates a strong correlation between the ER and the PR, whilst the ER and the PR are negatively associated with grade irrespective of the type of assessment employed. No association was evident between the ER or PR expression and histological subtype, suggesting that the automated approach is not disrupted by the clinical heterogeneity of breast cancer.

To further validate the algorithm, the prognostic power of the ER and PR expression as determined by image analysis was compared with that derived from standard pathological assessment. Cox univariate regression analysis was employed to compare OS based on manual scores for the ER and the PR versus OS obtained from automated analysis. Continuous automated assessment of the ER and PR expression was dichotomised using a threshold of 10%. Cox regression univariate analysis was performed on both cohorts to assess the difference in the RFS, OS and BCSS between ER-negative, PR-negative (< 10%) samples and ER-positive, PR-positive (>10%) samples using both manual pathologist-based assessment and automated image analysis. Table 2 summarises our findings in terms of univariate analysis of OS based on manual and automated analysis; the table demonstrates there is no significant difference in respect to hazard ratios (HRs) using either manual or automated analysis in both cohorts.

**Table 2:** Cox univariate regression comparing the random forest clustering (RFC) thresholds for the oestrogen receptor (ER) and the progesterone receptor (PR) with both the manual analysis and the 10% cutoff thresholds of the quantitative data

	Manual analysis threshold			10% automated threshold			RFC threshold		
			
	HR	95% CI	*P *value	HR	95% CI	*P *value	HR	95% CI	*P *value
Cohort I (*n* = 179)									
ER									
OS	0.259	0.127 to 0.530	0.001	0.154	0.067 to 0.355	0.002	0.235	0.116 to 0.473	0.001
PR									
OS	0.307	0.158 to 0.597	0.001	0.361	0.183 to 0.712	0.01	0.386	0.197 to 0.756	0.02

Cohort II (*n* = 564)									
ER									
OS	0.62	0.465 to 0.826	0.006	0.68	0.509 to 0.907	0.009	0.631	0.480 to 0.830	0.001
BCSS	0.677	0.494 to 0.928	0.006	0.664	0.492 to 0.898	0.008	0.592	0.445 to 0.787	< 0.001
RFS	0.714	0.540 to 0.943	0.009	0.797	0.603 to 1.054	0.11	0.701	0.538 to 0.914	0.009
PR									
OS	0.647	0.483 to 0.867	0.004	0.719	0.538 to 0.961	0.026	0.705	0.524 to 0.947	0.020
BCSS	0.602	0.445 to 0.814	0.001	0.7	0.518 to 0.947	0.020	0.673	0.495 to 0.916	0.012
RFS	0.73	0.549 to 0.972	0.03	0.797	0.603 to 1.053	0.790	0.791	0.593 to 1.054	0.109

HR, hazard ratio; CI, confidence interval; OS, overall survival; BCSS, breast cancer-specific survival; RFS, recurrence-free survival.

### Automatic determination of the optimal threshold for survival analysis based on ER and PR expression

Based on these findings we proceeded to use random forest clustering (RFC) in an attempt to identify new prognostic subgroups following quantitative assessment of the ER and the PR. RFC is an unsupervised strategy that has been used to profile tumours based on TMA data [12,13] and has been previously used by our group to identify new prognostic subgroups based on automated analysis of IHC data [10]. To this end, Cohort I was used as a training set and Cohort II was used as a validation set.

**Figure 2:**
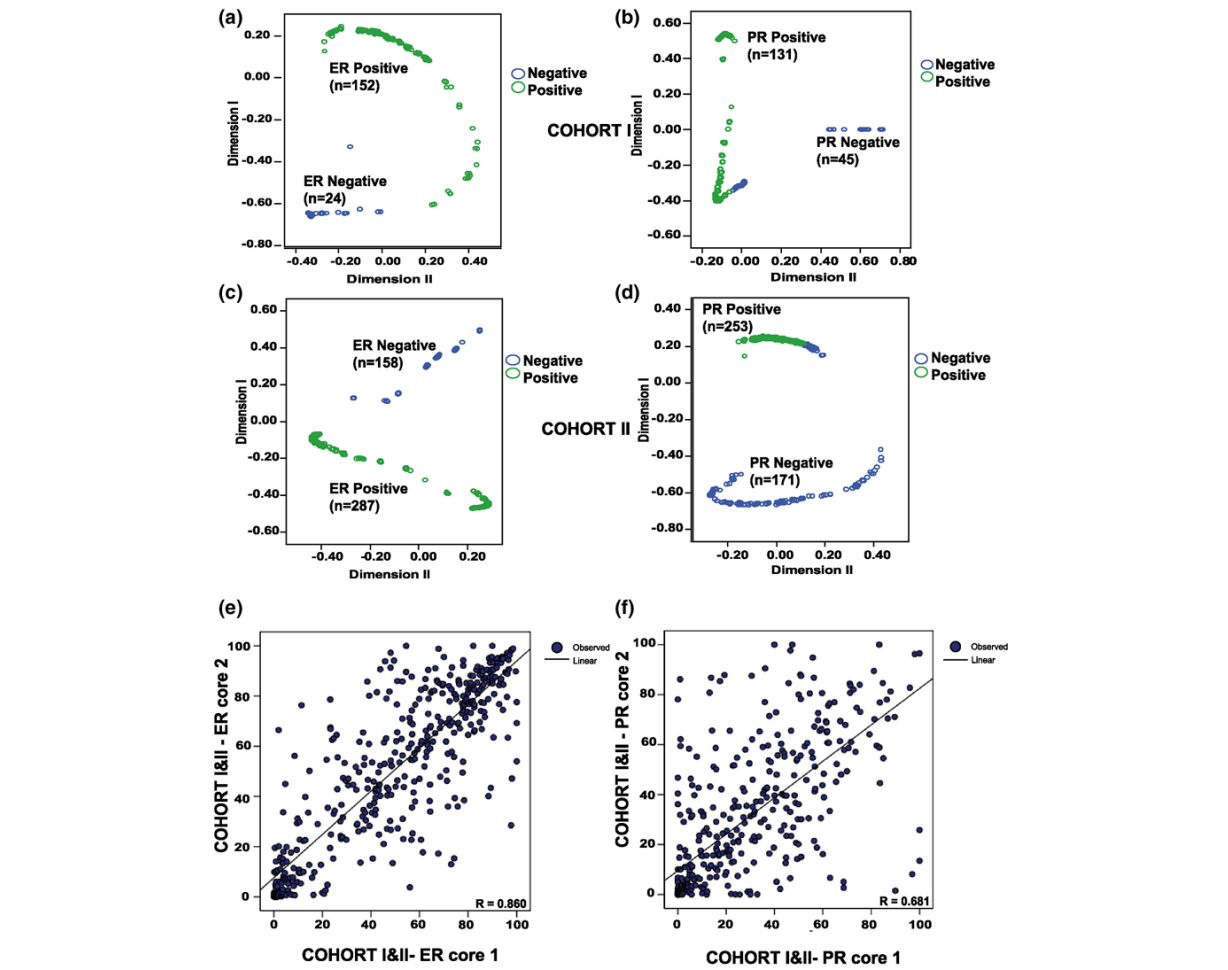
**Threshold identification and validation for oestrogen receptor and progesterone receptor data, and marker heterogeneity assessment**. Random forest clustering (RFC) clusters generated using automated quantitative oestrogen receptor (ER) and progesterone receptor (PR) data in Cohort I (test set): negative cluster and positive cluster for **(a) **the ER and **(b) **the PR. **(c) **ER RFC and **(d) **PR RFC in Cohort II (validation cohort). **(e) **Scatter plot demonstrating strong correlation between duplicate ER cores, indicating a homogenous pattern of expression. **(f) **Scatter plot showing weaker correlation between duplicate PR cores, indicating a more heterogeneous pattern of expression.

RFC was performed on the continuous ER and PR expression values as determined by image analysis. This procedure revealed two distinct clusters for ER and PR data in both cohorts (Figure 2a to 2d). RFC performed on Cohort I revealed an ER-negative cluster (*n* = 24) and an ER-positive cluster (*n* = 152); similar results were seen for the PR (Figure 2a, b). In Cohort II, the ER-negative cluster (*n* = 158) consisted of a group of patients with low levels of ER expression (mean = 3%, standard error of the mean = 0.1%), while patients within the ER-positive cluster (*n* = 287) had high levels of ER expression (mean = 51%, standard error of the mean = 2%) (Figure 2c, d). Likewise for the PR, the negative cluster (*n* = 171) consisted of a group of patients with low levels of PR expression (mean = 1%, standard error of the mean = 0.1%) and the positive cluster (*n* = 253) had high levels of PR expression (mean = 53%, standard error of the mean = 1%).

These data were, therefore, dichotomised based on the distribution of the ER and the PR within each cluster, using a threshold of 7% for the ER and of 5% for the PR to identify individual clusters. The OS and RFS were examined in both cohorts based on the thresholds identified following RFC analysis. Based on these thresholds, 9% (*n* = 2) of manually assessed ER-negative tumours would be classified as ER-positive in Cohort I and 11% (*n* = 15) of manually assessed ER-negative tumours would be classified as ER-positive in Cohort II. In Cohort I, the threshold as determined by RFC for the ER was associated with a similar HR for OS to that of the manual analysis and a 10% automated threshold. In Cohort II, however, RFC clusters were associated with a lower HR as compared with the 10% cutoff level based on automated or manual analysis for OS, RFS and BCSS (Table 2). A 5% cutoff threshold for the PR was associated with similar HRs to manual analysis; however, an automated 10% threshold as determined by image analysis was not associated with a significant outcome. The manually assessed ER and PR status in Cohort II was then compared with the RFC data, and revealed 13 patients (3%) who would not receive tamoxifen based on current manual analysis.

To investigate the true prognostic potential of a 7% cut-off for the ER and a 5% cut-off for the PR, we analysed RFS in the untreated arm of Cohort II. This analysis revealed that manual analysis using a 10% cutoff level was associated with a HR of 0.64 (95% confidence interval = 0.46 to 0.81, *P *< 0.001); however, a 7% threshold as determined following RFC was associated with a lower HR and narrower confidence intervals (HR = 0.59, 95% confidence interval = 0.44 to 0.77, *P *< 0.001). This would suggest that a 7% cutoff threshold may potentially be a stronger prognostic indicator in premenopausal women. The 5% cutoff level for the PR was not associated with an improved HR as compared with manual analysis, which could be attributed to the heterogeneous expression pattern of this protein (discussed below).

### Automated analysis of ER and PR expression and predicting tamoxifen response

Having demonstrated the prognostic benefit of a 7% threshold for the ER in the untreated arm of Cohort I, we proceeded to use the treated arm to investigate any relationship to tamoxifen response. This investigation revealed a similar effect of 2 years of tamoxifen treatment on the ER-positive and PR-positive cohort of patients as determined by RFC, compared with manual analysis. As expected, no treatment effect was evident in ER-negative patients or PR-negative patients as determined by RFC (data not shown). The effect of tamoxifen treatment was also examined using Cox regression analysis, which revealed that a 7% cutoff threshold for the ER was associated with improved RFS (HR 0.579, 95% confidence interval = 0.384 to 0.872, *P *= 0.009) compared with the manual or automated 10% thresholds. A similar effect was not evident for the PR (Table 3).

**Table 3:** Cox univariate regression of recurrence-free survival in the treated arm of Cohort II

	Cohort II treated arm (*n* = 276)					
	
	Oestrogen receptor			Progesterone receptor		
		
	HR	95% CI	*P *value	HR	95% CI	*P *value
Manual analysis	0.622	0.413 to 0.936	0.023	0.566	0.368 to 0.869	0.009
Automated 10% threshold	0.666	0.434 to 1.022	0.063	0.64	0.534 to 0.955	0.04
Automated clustering	0.579	0.384 to 0.872	0.009	0.67	0.431 to 1.043	0.07

HR, hazard ratio; CI, confidence interval.

### ER and PR core heterogeneity

To identify the reasons underlying our different results for the ER and for the PR, we compared the pattern of expression of both markers in the duplicate cores. TMAs are often criticised due to their apparent inability to account for tumour heterogeneity, which can partially be overcome by increased sampling. Automated analysis allows for a systematic evaluation of tumour heterogeneity between individual cores.

To assess the level of intra-tumour heterogeneity of ER and PR expression as defined by image analysis, we analysed the linear relationship between ER and PR duplicate cores (Figure 2e, f). By examining the linear relationship between ER (*n* = 639) and PR (*n* = 622) duplicate cores in both cohorts, higher linear regression coefficients were seen for the ER (*R *= 0.860) than for the PR (*R *= 0.681). This observation would suggest that duplicate cores from the same tumour have similar levels of ER expression as determined by image analysis, thus indicating the homogeneous nature of ER staining, whilst PR expression would be associated with a more heterogeneous pattern of expression. It should be noted that this does not indicate that TMA-based analysis of PR expression is not valid, and an excellent correlation between automated and manual assessment of PR expression was evident in this study (described above).

## Discussion

The purpose of the present study was to develop an unsupervised algorithm for automated quantification of IHC-determined nuclear protein expression in breast tumour specimens. In the current article we present data on the automated quantification of the ER and the PR in 743 primary breast tumours using such an algorithm. Our approach differs from other commercially available packages in that it does not require prior data for training purposes, and it uses a novel, fully automated approach to isolate tumour cells from stromal tissue and lymphocytic infiltrate. A good counterstain is helpful; however, it does not have to be specifically controlled for automated analysis. Heterogeneity in respect to counterstaining is handled in our approach by using a particular colour space (CIE Luminance U-chromatic component 1 V-chromatic component 2) combined with different morphological image operations. Such an approach allows for the identification of negative tumour nuclei from stromal elements, thus allowing for a more precise determination of protein expression. The accuracy of this approach was evident from the excellent correlation seen between automated quantification and manual analysis (Figure 1).

The first attempts to use automated methods to quantify protein expression, as assessed by IHC, were undertaken almost two decades ago [14,15]. Until recently, however, the image quality and analysis software did not permit high-throughput automated assessment of histological slides. A number of groups have now published data on the automated assessment of the ER and the PR in breast cancer [16-18]. In general, these studies are in agreement with the data presented in the current study in relation to describing an excellent correlation between manual and automated analysis. The studies by Turbin and colleagues and by Gokhale and colleagues describe commercially available algorithms that require specific input and training by a pathologist [16,17]. The algorithm described in the current study differs from these approaches in that it is entirely unsupervised and does not require any *a priori *data. Other approaches to the quantification of the ER and the PR described in the literature have used antibody-conjugated fluorophores and fluorescent microscopy systems [19,20]. This method, however, requires users to perform specific and complex staining protocols, which may not be suitable for a busy diagnostic service.

There has been much discussion in the literature regarding the optimal thresholds for the ER and/or the PR when identifying patients who will benefit from anti-endocrine therapy [20-23]. Currently, a threshold of 10% positive cells is commonly used to predict a patient's response to adjuvant hormonal therapy. Such a cutoff threshold can lead to significant intra-observer variability. One of the aims of the present study was to attempt to validate currently used thresholds for the ER and the PR, as well as to find new thresholds, using an unsupervised clustering approach. To this end, we were able to utilise a cohort of patients who had participated in a prospective randomised control trial comparing tamoxifen treatment with no adjuvant treatment [2]. The untreated arm of the trial was used to evaluate the prognostic value of new cutoff thresholds, while the treated arm was used to evaluate the predictive power of such thresholds.

RFC, an approach that has been used previously by our group [10], was utilised to identify new thresholds for survival analysis. This approach identified optimal thresholds of 7% for the ER and 5% for the PR. Interestingly, the 7% threshold for the ER was associated with a similar outcome in Cohort I and an improved outcome when compared with a manual 10% cutoff level in Cohort II (Table 2). This effect was not evident for the PR.

The true prognostic power of a 7% threshold was investigated in the untreated arm of Cohort II, and this confirmed our findings regarding both markers – specifically, ER manual analysis using a 10% cutoff threshold was associated with a HR of 0.64 (95% confidence interval = 0.46 to 0.81, *P *< 0.001). A 7% threshold as determined following RFC, however, was associated with a lower HR and narrower confidence intervals (HR = 0.59, 95% confidence interval = 0.44 to 0.77, *P *< 0.001), Finally, we examined the ability of our clusters to predict the tamoxifen response in a premenopausal cohort from a randomised control trial. This demonstrated that the RFC-based approach was associated with better outcomes, indicating that this approach would be a more accurate predictor of tamoxifen response (Table 3). This observation suggests that 10% is a suboptimal threshold for the ER when predicting the tamoxifen response. What should also be acknowledged, however, is that a 7% cutoff threshold for manual analysis is perhaps not feasible and would lead to an increase in both intra-observer and inter-observer variability.

These data also confirm previously published findings describing the heterogeneous pattern of expression for the PR [20]. One of the arguments against TMA-based approaches is that they do not give a true representation of proteins that display such heterogeneity in expression terms [24,25]. In the present study we have used an image analysis approach to address marker heterogeneity. If TMAs are to be used for high-throughput biomarker validation in the future, an ability to identify proteins exhibiting such varied expression patterns would be extremely helpful and avoid the reporting of potentially biased data. The method would also allow investigators to proceed, in a systematic manner, onto full-face sections at an earlier stage in the validation process. Our approach revealed that the ER displayed a much more homogeneous pattern of expression than the PR (Figure 1g, h). Our data are in agreement with other studies. For example, in a review of 5,993 cases, Nadji and colleagues demonstrated that the ER displayed a homogeneous pattern of expression in 92% of cases, whilst the PR displayed a heterogeneous pattern of expression in 21% of cases [26]. We believe this observation may indicate why our results for the PR were not as consistent as those seen for the ER; however, this does not preclude TMA-based analysis of PR expression.

## Conclusions

We have successfully developed and validated a novel approach to quantify nuclear protein expression in breast tumours using image analysis of IHC data. Moreover, we have validated 7% as the optimal cutoff threshold for the ER, by utilising two independent cohorts comprising a total of over 700 patients. Our data would also suggest that image analysis approaches may provide more sensitive measures of predicting tamoxifen response, whereby a 7% cutoff threshold predicts improved response to tamoxifen. Based on our findings in the present study, 13 patients (3%) who would not currently receive tamoxifen would have benefited from the drug. One potential weakness of the current study is that it was based on a TMA platform. Therefore, it is planned to proceed to apply this technology to full-face sections derived from breast biopsies and resections. This would allow the algorithm to be utilised in routine pathological practice.

There is an urgent need for new prognostic and predictive assays that would allow for improved patient stratification in breast cancer. Whilst a huge emphasis has been placed on DNA microarrays as the basis of such new tests [27], it is now becoming obvious that IHC surrogates may be a more appropriate clinical assay than gene expression-based platforms in the future [28]. As demonstrated by Nielsen and colleagues and by Careyand colleagues, it is possible to identify molecular subgroups (luminal A, luminal B, basal and Her2) using a small number of IHC markers [29,30]. Likewise, Ring and colleagues have reported on a novel panel of five antibodies that can predict outcome in ER-positive tumours [31]. More recently, Crabb and colleagues described a novel eight-marker, IHC-based, prognostic test for patients with advanced lymph-node-positive (>4 positive lymph nodes) disease [32]. These findings combined with large-scale antibody-based-proteomics resources, such as the Human Protein Atlas programme [33], will most probably lead to an increased use of multiplex IHC assays as both predictive and prognostic tests. The use of automated algorithms such as the one described in the present paper can only help to advance this process, whilst the use of novel approaches such as RFC may lead to the identification of new prognostic and predictive subgroups, allowing for tailored treatment regimens for individual patients – also known as personalised medicine.

## Abbreviations

BCSS: breast cancer-specific survival; DAB: 3,3'-diaminobenzidine; ER: oestrogen receptor; HR: hazard ratio; IHC: immunohistochemistry; OS: overall survival; PR: progesterone receptor; RFC: random forest clustering; RFS: recurrence-free survival; TMA: tissue microarray.

## Competing interests

ER, DJB and WMG were co-inventors of a patent application describing this method for image analysis purposes, lodged in Ireland on 4 December 2007. The remaining authors declare that they have no other competing interests.

## Authors' contributions

ER and DJB contributed equally to the present article. ER designed the algorithm and performed the statistical analysis. DJB performed the statistical analysis, designed the study and drafted the manuscript. PH validated the algorithm. EWK conceived the study and drafted the manuscript. AHM conceived the study and drafted the manuscript. GL provided the TMAs and drafted the manuscript. MJD conceived the study and drafted the manuscript. KJ provided the TMAs, performed the manual analysis of the ER and PR, and drafted the manuscript. WMG conceived the study, participated in its design and coordination, and helped draft the manuscript. All authors read and approved the final manuscript.

## Supplementary Material

Additional file 1 Word file containing a detailed description of each step of the automated image analysis approach used to quantify the ER and PR status. Click here for file

Additional file 2 Adobe file containing figures outlining the algorithm. Click here for file
