# Supplementary material for: Novel image analysis approach for quantifying expression of nuclear proteins assessed by immunohistochemistry: application to measurement of oestrogen and progesterone receptor levels in breast cancer
Source: Breast Cancer Res. 2008 Oct 23;10(5):R89. doi: 10.1186/bcr2187 (PMC2614526; doi:10.1186/bcr2187)
Supplement: Additional file 1 — Word file containing a detailed description of each step of the automated image analysis approach used to quantify the ER and PR status. [file bcr2187-S1.doc]

Supplementary Data:

The algorithm described in this paper proceeds in five steps. Figure 1A in Additional data file 2 is a schematic representation of these steps. Figure 1A in Additional data file 2 illustrates the graphical output at the end of each step.

1. Separating DAB from Haematoxylin.

Figure 2A in Additional data file 2 is a schematic representation of the methods employed by the algorithm in separating DAB from Haematoxylin. The main steps are:

- 1. For the purpose of separating DAB from Haematoxylin, images are transformed into the LUV colour space and LUV values for both DAB (DAB_C) and Haematoxylin (H_C) are defined using Matlab (Mathworks).
  2. DAB and Haematoxylin are then separated from the slide background and the connective tissue using the thresholds calculated above (DAB_C and H_C). Figure 2B in Additional data file 2 demonstrates the resulting images following the stain separation process.

1. Segmentation of DAB positive and DAB negative nuclear patterns.

Figure 2A in Additional data file 2 illustrates how the algorithm then proceeds to segment all nuclei present. Following the extraction of DAB and Haematoxylin, individual tumour nuclei are demarcated from surrounding non-specific staining. The same procedure is utilised for both DAB-positive and DAB-negative nuclei and the steps are described below.

- 1. The LUV values of either Haematoxylin or DAB from the original image are used to segment the positive and negative nuclei.

- 1. Mean shift segmentation followed by image morphological operations (i.e. dilation and erosion) are used to identify specific nuclear signal from surrounding non-specific DAB staining, allowing for better demarcation of nuclei.
  2. Watershed segmentation is then used to identify DAB-positive nuclei. Supplemental Fig. 3B shows the result of the final identification of the DAB-positive nuclei.

1. Learning of the morphological and spatial descriptors of the DAB-positive nuclear patterns.

The image processing toolbox is used to find the X,Y position of the nuclei’s centre.

- 1. Each nucleus is further described by its area, length of the minor and major axis of the smallest ellipse containing the nuclei and the distance to its neighbour’s nuclei. Figure 3B in Additional data file 2 illustrates the geometrical definition of each of these parameters.
  2. The next step is to develop a mathematical representation of the morphological pattern of DAB-positive nuclei. The data extracted from step 3.1 is used to cluster the DAB-positive nuclei in two clusters, namely cluster 1 (nuclei close to each other) and cluster 2 (nuclei far from each other).

1. Selection of tumour DAB-negative nuclear patterns.

Once the DAB-negative nuclei are identified, a similar methodology is employed, whereby the nuclear pattern is mathematically delineated. The DAB-negative nuclear pattern contains different types of nuclei, i.e. tumour nuclei, stromal cells and lymphocytic infiltrate. The approach adopted proceeds by eliminating all non-tumour nuclei. Figure 4A in Additional data file 2 illustrates the approach of the elimination of non-relevant nuclear patterns and Figure 4B in Additional data file 2 demonstrates the output of the algorithm.

1. Final assessment of biomarker expression.

The last step is the evaluation of mean intensity of DAB staining and the percentage of DAB positive nuclei. DAB staining intensity is calculated based on the mean RGB values of all pixels part of nuclei isolated in step 2. The final percentage of DAB-positive tumour nuclei is assessed as the ratio of the total number of DAB-positive nuclei and the total number of both negative and positive DAB tumour nuclei.
