# Supplementary material for: Novel image analysis approach for quantifying expression of nuclear proteins assessed by immunohistochemistry: application to measurement of oestrogen and progesterone receptor levels in breast cancer
Source: Breast Cancer Res. 2008 Oct 23;10(5):R89. doi: 10.1186/bcr2187 (PMC2614526; doi:10.1186/bcr2187)
Supplement: Additional file 2 — Adobe file containing figures outlining the algorithm. [file bcr2187-S2.pdf]

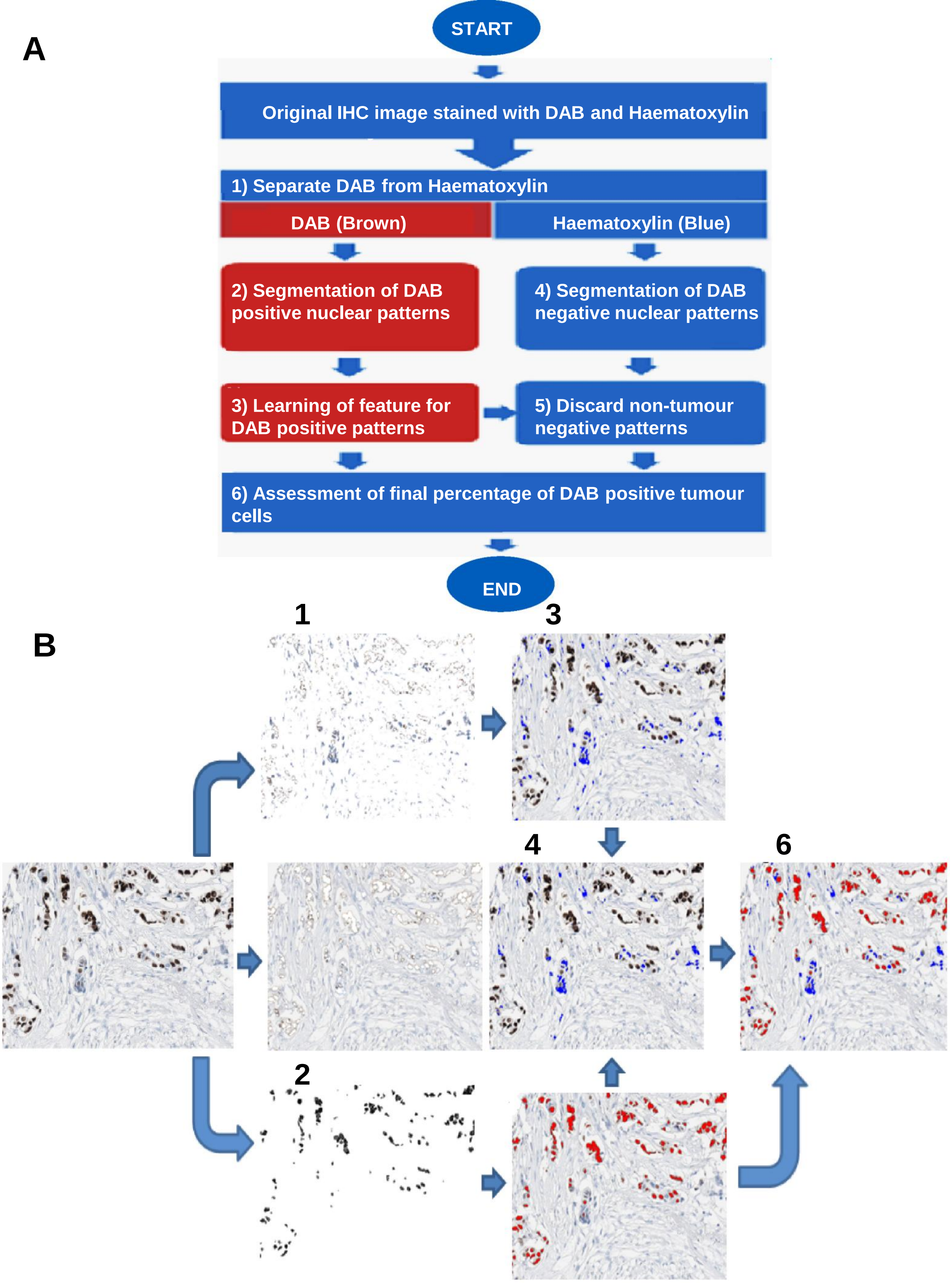
A
START
Original IHC image stained with DAB and Haematoxylin
1) Separate DAB from Haematoxylin
DAB (Brown)
Haematoxylin (Blue)
2) Segmentation of DAB positive nuclear patterns
4) Segmentation of DAB negative nuclear patterns
3) Learning of feature for DAB positive patterns
5) Discard non-tumour negative patterns
6) Assessment of final percentage of DAB positive tumour cells
END
B
1
3
4
6
2

Figure 1

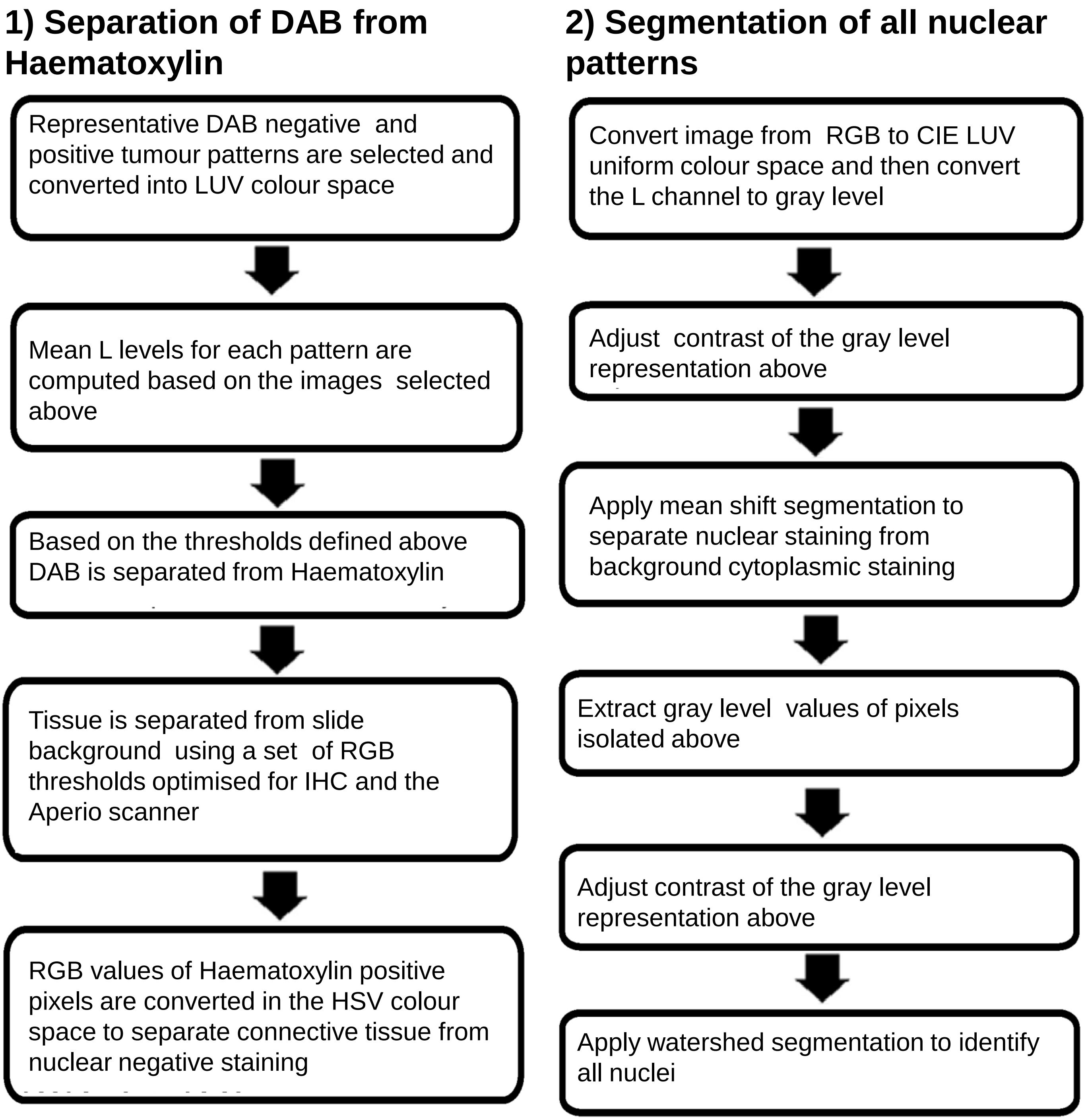
1) Separation of DAB from Haematoxylin
Representative DAB negative and positive tumour patterns are selected and converted into LUV colour space
Mean L levels for each pattern are computed based on the images selected above
Based on the thresholds defined above DAB is separated from Haematoxylin
Tissue is separated from slide background using a set of RGB thresholds optimised for IHC and the Aperio scanner
RGB values of Haematoxylin positive pixels are converted in the HSV colour space to separate connective tissue from nuclear negative staining
2) Segmentation of all nuclear patterns
Convert image from RGB to CIE LUV uniform colour space and then convert the L channel to gray level
Adjust contrast of the gray level representation above
Apply mean shift segmentation to separate nuclear staining from background cytoplasmic staining
Extract gray level values of pixels isolated above
Adjust contrast of the gray level representation above
Apply watershed segmentation to identify all nuclei

Figure 2

Extraction of DAB positive nuclei and their morphological and spatial properties

A

1) Find for each DAB positive nuclei :
- X, Y position in the image
- Area
- The small and big axis of the outbound ellipse

↓

Measure the distance between each nuclei and its neighbours

↓

2) Nuclei-to-nuclei distances are clustered in two groups:
- Distances of nuclei belonging to nuclei part of the same tumour gland (cluster D1)
- Distances of isolated nuclei or nuclei part of different tumour glands (cluster D2)

↓

Identify nuclei part of cluster D1 and discard nuclei in cluster D2

↓

3) From the nuclei identified above, the following features are extracted:
Mean area (MAP), mean length of small and big axis, mean (MDN) and max nuclei-to-nuclei distance, total number of nuclei and mean intensity of DAB positive nuclei

B

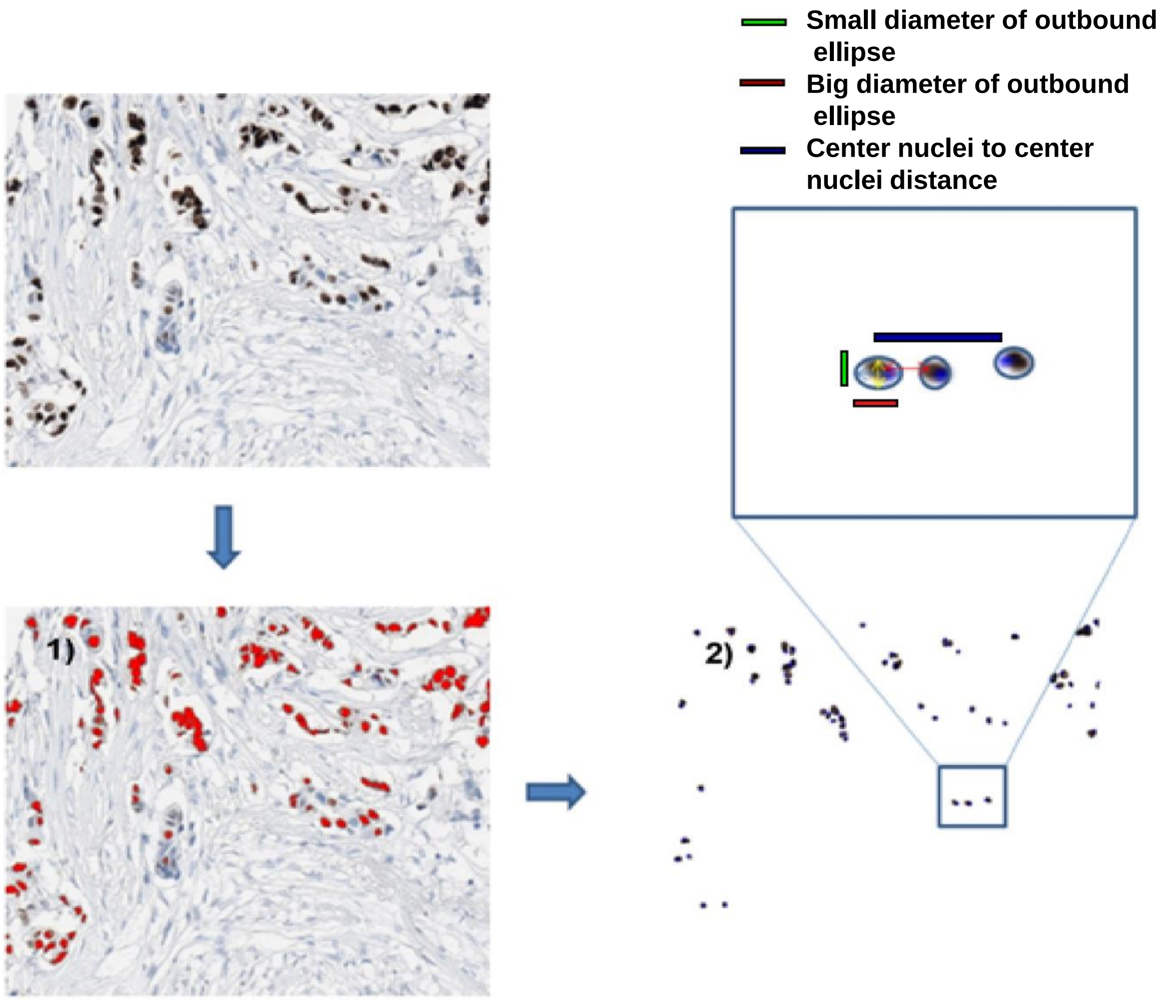

Figure 3

Extraction of DAB tumor negative nuclei

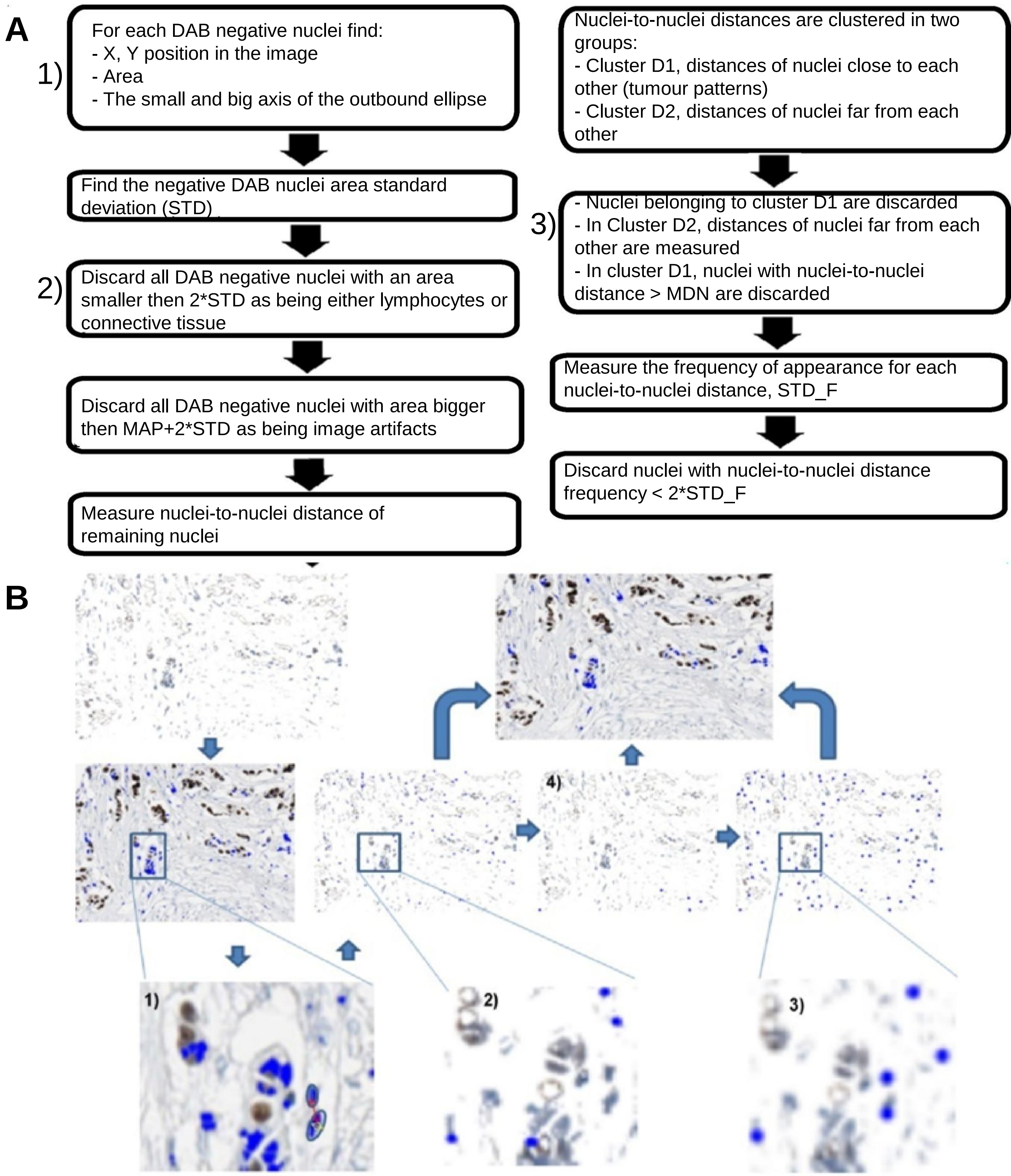

Figure 4
